# Efficacy of Hydroxychloroquine and Tocilizumab in Patients With COVID-19: Single-Center Retrospective Chart Review

**DOI:** 10.2196/21758

**Published:** 2020-09-01

**Authors:** Sohaib Roomi, Waqas Ullah, Faizan Ahmed, Soban Farooq, Usama Sadiq, Asad Chohan, Munnam Jafar, Maryum Saddique, Shristi Khanal, Robert Watson, Margot Boigon

**Affiliations:** 1 Jefferson Health Abington Abington, PA United States; 2 King Edward Medical University Lahore Pakistan; 3 Newark Beth Israel Medical Center Newark, NJ United States; 4 Einstein Medical Center Philadelphia, PA United States

**Keywords:** COVID-19, hydroxychloroquine, tocilizumab

## Abstract

**Background:**

During the initial phases of the COVID-19 pandemic, there was an unfounded fervor surrounding the use of hydroxychloroquine (HCQ) and tocilizumab (TCZ); however, evidence on their efficacy and safety have been controversial.

**Objective:**

The purpose of this study is to evaluate the overall clinical effectiveness of HCQ and TCZ in patients with COVID-19. We hypothesize that HCQ and TCZ use in these patients will be associated with a reduction in in-hospital mortality, upgrade to intensive medical care, invasive mechanical ventilation, or acute renal failure needing dialysis.

**Methods:**

A retrospective cohort study was performed to determine the impact of HCQ and TCZ use on hard clinical outcomes during hospitalization. A total of 176 hospitalized patients with a confirmed COVID-19 diagnosis was included. Patients were divided into two comparison groups: (1) HCQ (n=144) vs no-HCQ (n=32) and (2) TCZ (n=32) vs no-TCZ (n=144). The mean age, baseline comorbidities, and other medications used during hospitalization were uniformly distributed among all the groups. Independent *t* tests and multivariate logistic regression analysis were performed to calculate mean differences and adjusted odds ratios with 95% CIs, respectively.

**Results:**

The unadjusted odds ratio for patients upgraded to a higher level of care (ie, intensive care unit) (OR 2.6, 95% CI 1.19-5.69; *P*=.003) and reductions in C-reactive protein (CRP) level on day 7 of hospitalization (21% vs 56%, OR 0.21, 95% CI 0.08-0.55; *P*=.002) were significantly higher in the TCZ group compared to the control group. There was no significant difference in the odds of in-hospital mortality, upgrade to intensive medical care, need for invasive mechanical ventilation, acute kidney failure necessitating dialysis, or discharge from the hospital after recovery in both the HCQ and TCZ groups compared to their respective control groups. Adjusted odds ratios controlled for baseline comorbidities and medications closely followed the unadjusted estimates.

**Conclusions:**

In this cohort of patients with COVID-19, neither HCQ nor TCZ offered a significant reduction in in-hospital mortality, upgrade to intensive medical care, invasive mechanical ventilation, or acute renal failure needing dialysis. These results are similar to the recently published preliminary results of the HCQ arm of the Recovery trial, which showed no clinical benefit from the use of HCQ in hospitalized patients with COVID-19 (the TCZ arm is ongoing). Double-blinded randomized controlled trials are needed to further evaluate the impact of these drugs in larger patient samples so that data-driven guidelines can be deduced to combat this global pandemic.

## Introduction

As of July 23, 2020, more than 4 million cases and 140,000 deaths from COVID-19 have been reported in the United States. There is currently no proven medical therapy for this disease except low-dose dexamethasone and remdesivir based on preliminary evidence with the mainstay of treatment being supportive care [1]. Multiple off-label and compassionate use therapies are currently being employed, targeting currently known pathophysiological mechanisms of this novel virus. Increasing social and economic devastation caused by COVID-19 has led the Federal Drug Administration (FDA) to issue emergency use authorizations (EUAs) for various drugs without proven benefits [2]. Although many of these drugs have revealed promising in vitro activity against the coronaviridae family, including SARS-CoV-2, the translation of these in vitro effects into clinical efficacy is a matter of debate. While, as physicians, we tend to assume that these drugs will do more good than harm when utilizing them as a last resort to severely ill patients, the fact remains that in the absence of randomized controlled trials, there is no way to reliably judge the impact of these medications. Fortunately, this situation is being remedied, with evidence emerging, initially from China, and more recently from trials in the United States and Europe.

Among others, hydroxychloroquine (HCQ) and the interleukin-6 (IL-6) inhibitor, tocilizumab (TCZ), became popular options to treat COVID-19. There is no concrete evidence supporting their use, and they were widely adopted across the world based on anecdotal data. Our hospital, following the guidelines of its parent enterprise, permitted the use of HCQ in COVID-19 patients who had respiratory insufficiency as indicated by low oxygen saturation. Similarly, TCZ was used for patients who met the criteria for cytokine release syndrome during the time frame of this study. The purpose of this study is to evaluate the overall clinical effectiveness of HCQ and TCZ in our hospital. We will compare our results to the preliminary results of the HCQ arm of the RECOVERY trial, which did not reveal a difference in 28-day mortality between the HCQ group and the usual care group [3]. In addition to mortality, we will evaluate other secondary endpoints and hypothesize that use of HCQ and TCZ will be associated with a reduction in the endpoints of an upgrade to the intensive care unit (ICU), need for invasive mechanical ventilation (IMV), acute renal failure necessitating dialysis, and reduction in D-dimer and C-reactive protein (CRP) on the 7th day of hospitalization.

## Methods

### Study Design and Participants

This retrospective cohort study included adult inpatients (≥18 years old) from Abington Hospital - Jefferson Health in the United States. All patients had a confirmed diagnosis of COVID-19 between March 1, 2020, and May 30, 2020. The study was approved by the Institutional Review Board, and the requirement for informed consent was waived by the Research Ethics Committee.

### Data Collection

All COVID-19 patients who were admitted to the hospital between March 1, 2020, and May 30, 2020, were included. Data were extracted from electronic medical records (Sunrise) using a standardized data collection form. All authors contributed to data retrieval and an independent author adjudicated any difference in interpretation between the data extractors. SARS-CoV-2 was detected in respiratory specimens (nasopharyngeal or throat swabs) by real-time qualitative polymerase chain reaction (RT-qPCR). Routine blood work included complete blood count, serum electrolytes, renal function test, coagulation profile, serum ferritin, CRP, D-dimer level, lactate dehydrogenase, and myocardial enzymes (troponin T) on presentation to the hospital and on day 7 of hospitalization. Baseline comorbidities, including hypertension (HTN), diabetes mellitus (DM), chronic kidney disease (CKD), chronic obstructive lung disease (COPD), and coronary artery disease (CAD), were also recorded. The criteria of discharge from the hospital after recovery included resolution of fever, absence of symptoms for at least 1 day, and substantial clinical or radiological improvement.

### Statistical Analysis

A chi-square (χ^2^) test was used for comparison of categorical data and Fisher exact test was adopted if the expected count in more than 20% cells was less than 5. Continuous variables were presented as means and standard deviations while categorical variables were reported in percentages and proportions. To quantify the association between the dichotomous categorical variables, an unadjusted odds ratio (uOR) was obtained using a Cochran-Mantel-Haenszel method. To explore the risk factors and gauge the impact of potential effect modifiers (covariates) on our endpoints (in-hospital mortality, ICU upgrade, IMV, dialysis, and inflammatory marker level), binomial and multinomial logistic regression models were applied. The differences in the baseline comorbidities (DM, HTN, CAD, CKD, and COPD) and medication use (HCQ, TCZ, remdesivir, therapeutic anticoagulation, and steroids) were accounted for to obtain an adjusted odds ratio (aOR) for all outcomes. For normally and abnormally distributed continuous data, an independent sample *t* test and Mann-Whitney *U* test were used, respectively. A one-way analysis of variance (ANOVA) was used to compare differences in the mean of continuous variables for multiple in-hospital complications. A two-sided α<.05 was considered statistically significant with corroborating inference from a 95% CI. Statistical analyses were performed using the SPSS software (version 25, IBM Corp).

## Results

### Demographics and Baseline Characteristics

**Figure 1:**
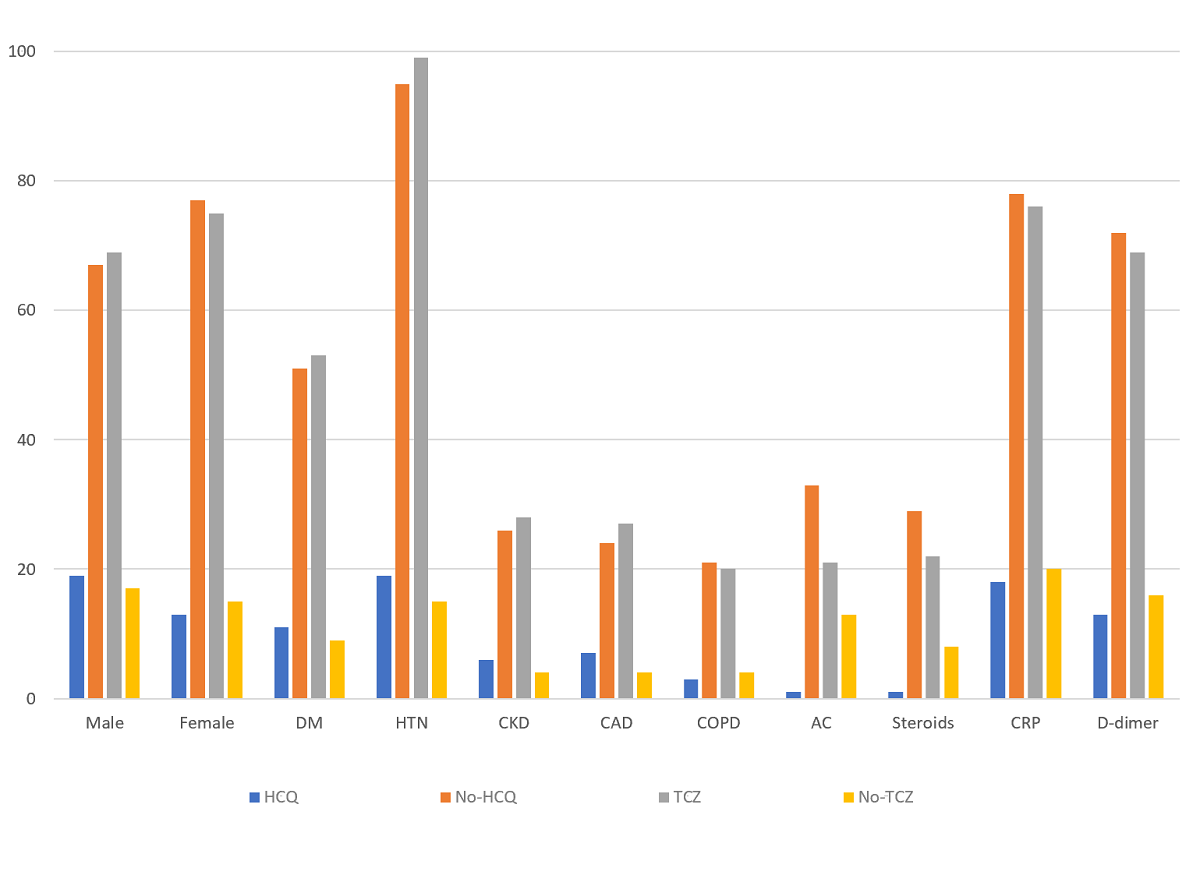
Baseline comorbidities and medication use in the hydroxychloroquine (HCQ) group, tocilizumab (TCZ) group, and control groups. The x-axis represents sex, comorbidities, medications, C-reactive protein (CRP), and D-dimer level at presentation; the y-axis represents the percentage of subjects. DM: diabetes mellitus; HTN: hypertension; CKD: chronic kidney disease; CAD: coronary artery disease; COPD: chronic obstructive pulmonary disease; AC: anticoagulation.

Our study population consisted of 176 patients who were hospitalized and had a confirmed case of COVID-19 infection. All patients were divided into two comparison groups: (1) HCQ (n=144) vs no-HCQ (n=32) and (2) TCZ (n=32) vs no-TCZ (n=144), respectively. Table 1 and Figure 1 depict the underlying comorbidities and other medications used during hospitalization in both comparison groups. The mean age in years for the HCQ and no-HCQ groups was 63.75 and 65.87 years, respectively (*P*=.55); for the TCZ and no-TCZ groups, it was 58.09 and 65.48 years, respectively (*P*=2.75). The most common underlying comorbidities in all the four groups were DM, HTN, CAD, CKD, and COPD. Common medications used during hospitalization included steroids, anticoagulants, HCQ, and TCZ. These underlying comorbidities and medications used during hospitalization were nonsignificantly different between the comparison groups (*P*≥.05). The detailed percentages of group-wise comorbidities and demographics are given in Table 1.

**Table 1:** Baseline characteristics of the included population across comparison groups.

Characteristic		No-HCQ^a^	HCQ	*P* value	No-TCZ^b^	TCZ	*P* value
Age (years), mean		65.87	63.75	.55	58.09	65.48	2.75
**Sex**				.17			.44
	Male, n (%)	66 (80.50)	16 (19.50)		23 (27.70)	60 (72.30)	
	Female, n (%)	75 (88.20)	10 (11.80)		13 (14.90)	74 (85.10)	
**Diabetes mellitus**				.72			.97
	No, n (%)	92 (85.20)	16 (14.80)		23 (21.10)	86 (78.90)	
	Yes, n (%)	49 (83.10)	10 (16.90)		13 (21.30)	48 (78.70)	
**Hypertension**				.24			.61
	No, n (%)	48 (80.00)	12 (20.00)		14 (23.30)	46 (76.70)	
	Yes, n (%)	93 (86.90)	14 (13.10)		22 (20.00)	88 (80.00)	
**Coronary artery disease**				.16			.78
	No, n (%)	114 (82.60)	24 (17.40)		30 (21.60)	109 (78.40)	
	Yes, n (%)	27 (93.10)	2 (6.90)		6 (19.40)	25 (80.60)	
**Chronic kidney disease**				.65			.56
	No, n (%)	114 (83.80)	22 (16.20)		28 (20.30)	110 (79.70)	
	Yes, n (%)	27 (87.10)	4 (12.90)		8 (25.00)	24 (75.00)	
**Chronic obstructive pulmonary disease**				.29			.56
	No, n (%)	119 (83.20)	24 (16.80)		32 (21.90)	114 (78.10)	
	Yes, n (%)	22 (91.70)	2 (8.30)		4 (16.70)	20 (83.30)	
**Steroids**				.39			.25
	No, n (%)	115 (83.30)	23 (16.70)		32 (22.90)	108 (77.10)	
	Yes, n (%)	26 (89.70)	3 (10.30)		4 (13.30)	26 (86.70)	
**Anticoagulation**				.32			.08
	No, n (%)	115 (85.80)	19 (14.20)		25 (18.40)	111 (81.60)	
	Yes, n (%)	26 (78.80)	7 (21.20)		11 (32.40)	23 (67.60)	

^a^HCQ: hydroxychloroquine.

^b^TCZ: tocilizumab.

### Odds Ratios of Outcomes

**Figure 2:**
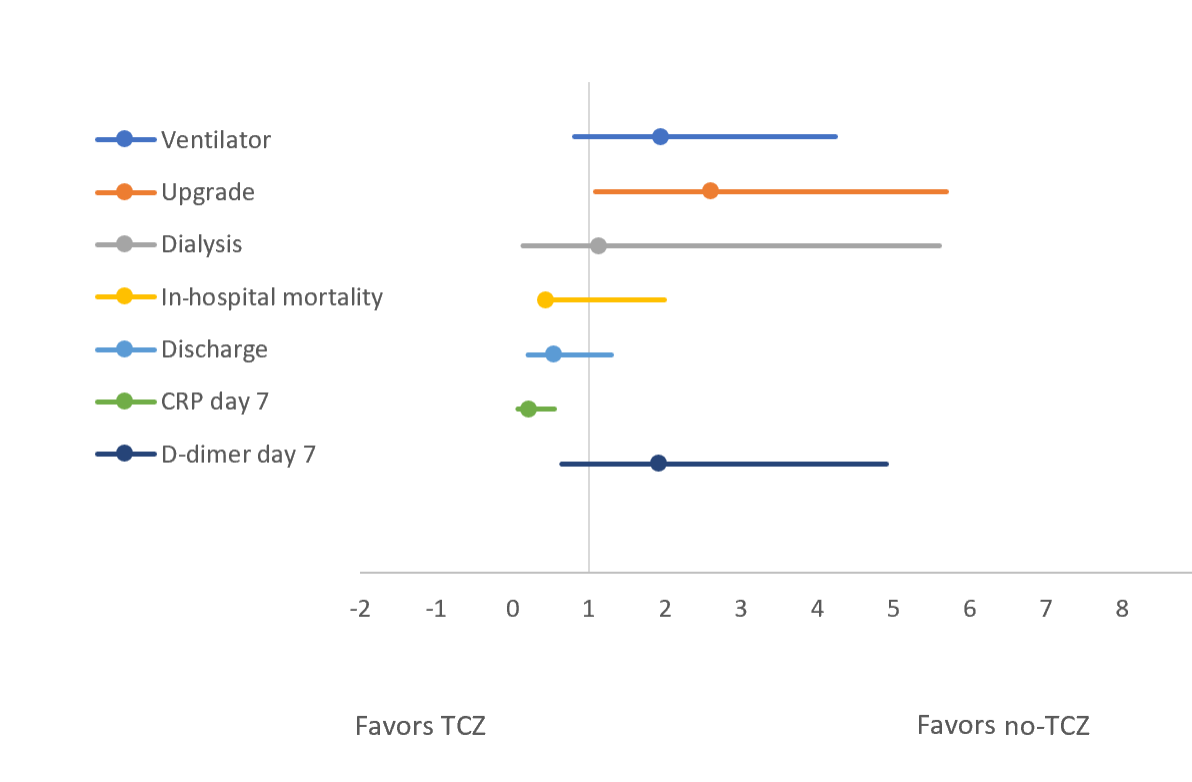
Forest plot comparing unadjusted odds of outcomes between the tocilizumab (TCZ) and no-TCZ groups. CRP: C-reactive protein.

**Figure 3:**
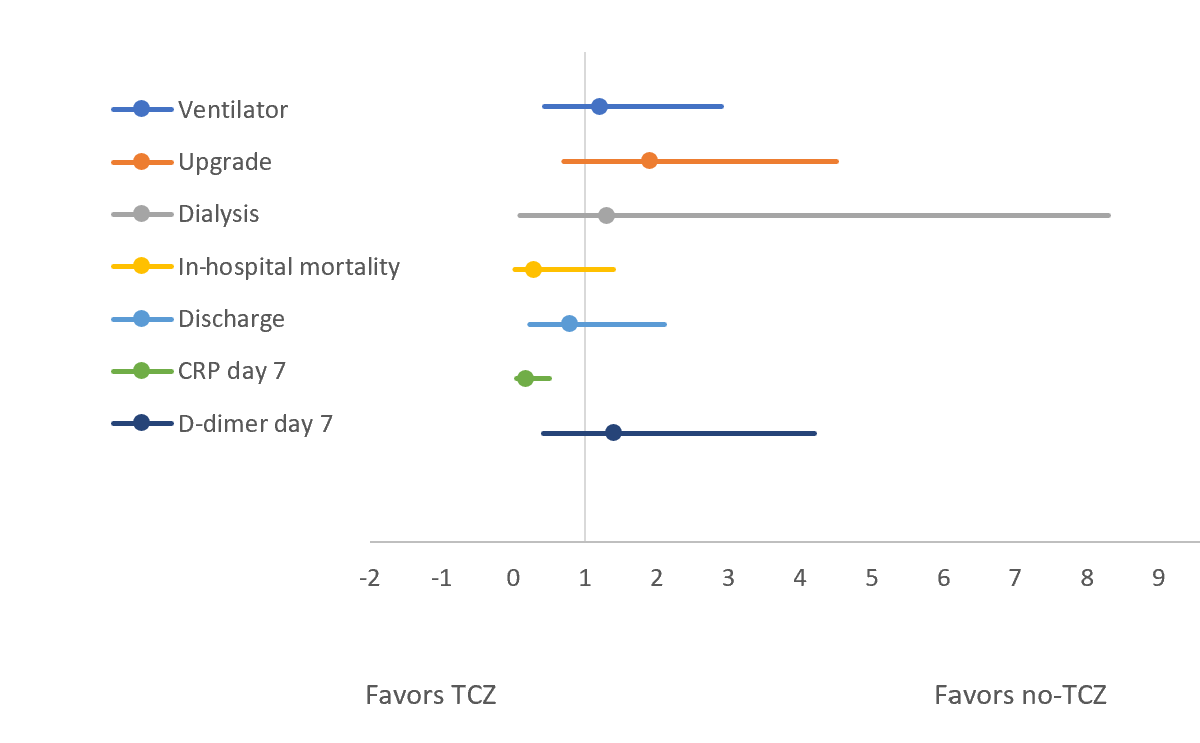
Forest plot comparing adjusted odds of outcomes between the tocilizumab (TCZ) and no-TCZ groups. CRP: C-reactive protein.

Table 2 and Figures 2 and 3 compare the above-mentioned outcomes between the TCZ group and the no-TCZ group. The unadjusted odds ratio for patients requiring an upgrade to the ICU was significantly higher in those who received TCZ compared to the control group (OR 2.6, 95% CI 1.19-5.69; *P*=.003). Similarly, patients who received TCZ had a significant reduction in CRP levels on day 7 of hospitalization compared to the control group (21% vs 56%, OR 0.21, 95% CI 0.08-0.55; *P*=.002). However, this reduction in the inflammatory markers did not translate into clinical benefits. There was no significant difference in the unadjusted odds of in-hospital mortality, IMV, acute renal failure necessitating dialysis, and discharge from the hospital after recovery between the two groups. The proportion of high D-dimer levels (>500 ng/dL) and elevated CRP (>100 ng/dL) on day 7 of hospitalization were also identical between the TCZ and no-TCZ groups. However, when we adjusted the observed odds ratios for baseline comorbidities, including DM, HTN, CKD, CAD, COPD, medications, use of anticoagulation at home, therapeutic anticoagulation during hospital stay, as well as steroid and HCQ use in the TCZ comparison group, the adjusted odds values were consistent with unadjusted odds ratios for all the outcomes. The exception was for an upgrade to medial ICU, where there was no difference between the TCZ group and the no-TCZ group. This is contrary to the unadjusted odds, which revealed more ICU upgrades in the TCZ group compared to the no-TCZ group (Table 2). The forest plots given in Figures 2 and 3 reveal the difference in unadjusted and adjusted odds between the TCZ group and the no-TCZ group.

**Table 2:** Tocilizumab (TCZ) regression analysis with outcome.

Outcome		TCZ, n	No TCZ, n	uOR^a^ (95% CI)	*P* value	aOR^b^ (95% CI)	*P* value
Invasive mechanical ventilation		47	31	1.94 (0.89-4.23)	.14	1.2 (0.49-2.9)	.67
Upgrade		50	28	2.6 (1.19-5.69)	.03	1.9 (0.80-4.5)	.14
Dialysis		6	6	1.13 ( 0.23-5.6)	.79	1.3 (0.21-8.3)	.76
Mortality		6	13	0.44 (0.97-1.99)	.43	0.28 (0.05-1.4)	.13
Discharge		25	38	0.54 (0.23-1.3)	.23	0.78 (0.28-2.1)	.64
**D-dimer**							
	Day 1	50	52	0.93 (0.43-2.01	.99	0.7 (0.31-1.7)	.47
	Day 7	77	64	1.92 (0.76-4.9)	.24	1.4 (0.52-4.2)	.45
**C-reactive protein**							
	Day 1	63	55	1.38 (0.63-3.04)	.55	1.26 (0.54-2.93)	.59
	Day 7	21	56	0.21 (0.08-0.55)	.002	0.17 (0.05-0.50)	.001

^a^uOR: unadjusted odds ratio.

^b^aOR: adjusted odds ratio.

**Figure 4:**
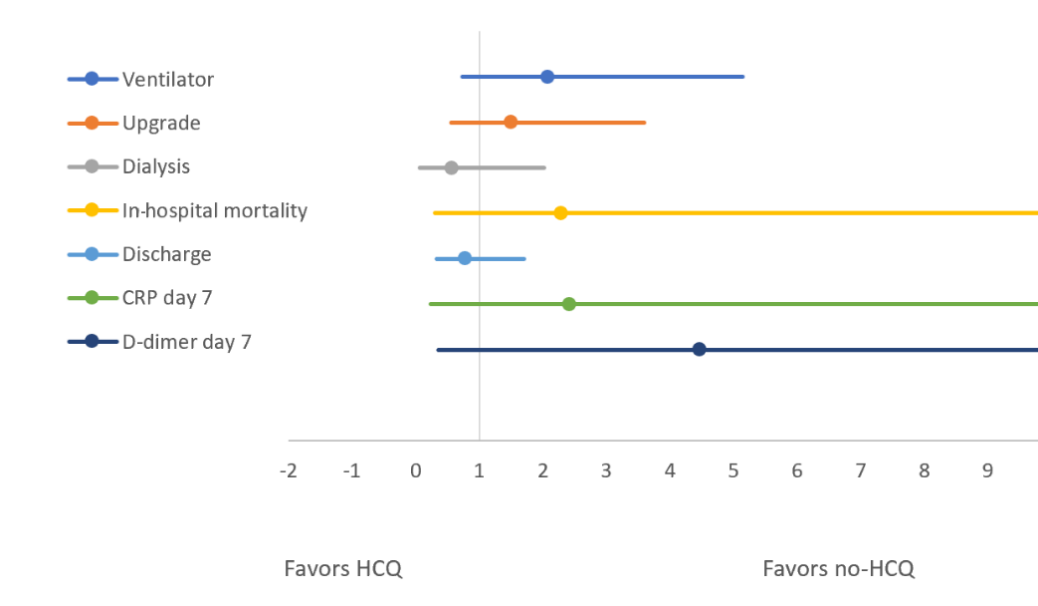
Forest plot comparing unadjusted odds of outcomes between the hydroxychloroquine (HCQ) and no-HCQ groups. CRP: C-reactive protein.

**Figure 5:**
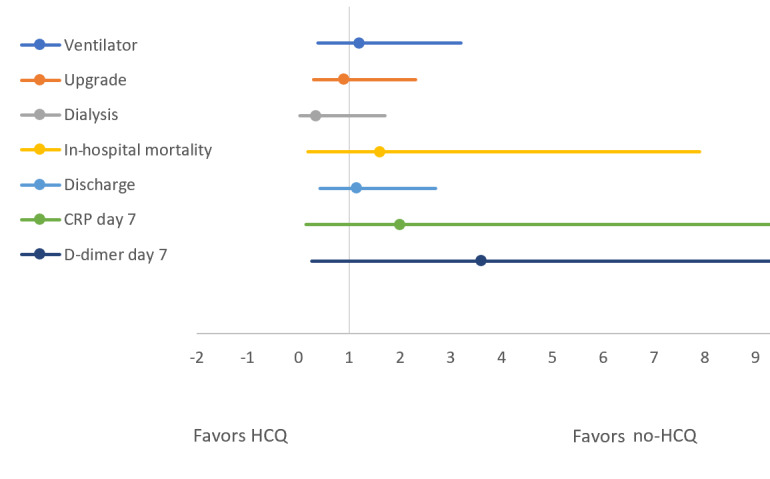
Forest plot comparing the adjusted odds of outcomes between hydroxychloroquine (HCQ) and no-HCQ groups. CRP: C-reactive protein.

Similarly, Table 3 and Figures 4 and 5 compare the above-mentioned outcomes between the HCQ group and the no-HCQ group. The use of HCQ in patients with COVID-19 was not associated with a significant improvement in any of the outcomes. The unadjusted odds ratio of in-hospital mortality, upgrade to ICU, IMV, acute renal failure needing dialysis, or discharge after recovery were identical between patients receiving HCQ or not, respectively. Similarly, the proportion of high D-dimer and CRP levels on day 7 of hospitalization was not significantly different between the HCQ and no-HCQ groups. As with the TCZ comparison group, a multivariate regression analysis was used to adjust the observed odds ratios for baseline comorbidities and medications including TCZ in the HCQ comparison group. The adjusted odds values were consistent with unadjusted odds ratios for all the outcomes as having been depicted by similar forest plots in Figures 4 and 5.

**Table 3:** Hydroxychloroquine (HCQ) regression analysis with outcome.

Outcome		HCQ, n	No HCQ, n	uOR^a^ (95% CI)	*P* value	aOR^b^ (95% CI)	*P* value
Invasive mechanical ventilation		37	22	2.08 (0.84-5.14)	.16	1.2 (0.46-3.2)	.68
Upgrade		33	25	1.5 (0.63-3.59)	.48	0.9 (0.35-2.3)	.84
Dialysis		5	9	0.57 (0.12-2.02)	.57	0.34 (0.06-1.7)	.19
Mortality		13	6	2.28 (0.5-10.3)	.43	1.6 (0.33-7.9)	.54
Discharge		35	41	0.78 (0.36-1.7)	.68	1.15 (0.48-2.7)	.74
**D-dimer**							
	Day 1	51	54	0.89 (0.37-2.10)	.95	0.8 (0.3-1.9)	.63
	Day 7	69	33	4.47 (0.77-25)	.18	3.6 (0.59-22.7)	.16
**C-reactive protein**							
	Day 1	55	62	0.75 (0.33-1.69)	.62	0.64 (0.27-1.51	.31
	Day 7	49	29	2.42 (0.45-12.95	.50	2.0 (0.33-12.8)	.44

^a^uOR: unadjusted odds ratio.

^b^aOR: adjusted odds ratio.

## Discussion

### Principal Findings

The purpose of this study is to evaluate the overall clinical effectiveness of HCQ and TCZ in our hospital. Our results revealed that both TCZ and HCQ had no role in improving hard clinical outcomes in patients with COVID-19 admitted to the hospital. Compared to patients in the control group, those who received either of these medications did not show a significant reduction in the rate of in-hospital mortality, upgrade to ICU, IMV, reduction in acute renal failure to the point of needing dialysis, or discharge from the hospital after recovery. Although the patients who received TCZ appeared to have a higher rate of ICU upgrade, this trend seemed to be driven by multiple comorbidities in the TCZ group, as evidenced by an identical adjusted odds ratio on multivariate analysis (Table 2; Figures 2 and 3). Partly contributing to this might be the higher use of TCZ in the sicker patients who fulfilled the criterion to receive the drug based on disease severity.

HCQ and TCZ, the major therapy for rheumatological diseases, have recently gained attention as one of the major cornerstone management approaches for COVID-19. HCQ is thought to work by inhibiting glycosylation of the host receptors, endosomal acidification, and proteolytic processing thereby, blocking viral entry into host cells [4-7]. TCZ, on the other hand is believed to counteract the misdirected immune response related to the COVID-19 cytokine storm [8]. Being a monoclonal antibody directed against IL-6, TCZ is thought to dampen the immune response and potentially reduce the adverse outcomes related to COVID-19.

A previous study by Xu et al [9] has shown a significant improvement in respiratory function (91% reduction in symptoms) and length of hospital stay in COVID-19 patients with a single dose of TCZ. However, that study was underpowered (n=21 patients) and had no control arm [9]. Similarly, Luo and colleagues [10] observed an 80% survival rate in patients receiving TCZ. Their study also was not followed up by a large-scale study and had several limitations. A recently published retrospective cohort study that included 544 patients admitted in different hospitals of Italy revealed that after adjustment for sex, age, recruiting center, duration of symptoms, and SOFA (sequential organ failure assessment) score, TCZ treatment was associated with a reduced risk of IMV or death (adjusted hazard ratio 0.61, 95% CI 0.40-0.92; *P*=.020) [11]. Our study consisted of 176 patients, and it demonstrated that there were no major clinical benefits to TCZ use in COVID-19 patients. A significant reduction of CRP levels on day 7 of hospitalization was observed in the TCZ group compared to the control group; yet this difference did not translate into clinical benefits in terms of a reduction in in-hospital mortality, medical ICU upgrade, or reduction in IMV (Figures 2 and 3). As mentioned in the study limitations below, a larger patient population and a randomized controlled design might have demonstrated a clinical benefit parallel to this reduction in CRP level. Among other ongoing randomized, double-blinded, controlled trials, the Oxford-based RECOVERY trial is also recruiting participants who meet the eligibility criteria into the TCZ arm of the trial [12].

Similarly, preliminary data from China reported that HCQ use was associated with a reduction in the viral load, duration of disease, and resolution of COVID-19 pneumonitis on imaging [13]. A small, nonrandomized, open-label French study consisting of 36 patients also reported significant reduction in the viral load in patients taking HCQ [14]. Major subsequent large-scale trials also reported its potential utility in reducing the need for IMV. However, the medical community was concerned regarding the potential cardiovascular adverse effects of off-label HCQ use. Despite all the controversies surrounding HCQ, its use prevailed in the earlier part of the pandemic, leading to stockpiling and shortage of HCQ in international markets, then followed by a swift decline in its use [15].

In our study, we systematically determined the impact of HCQ on the hard clinical outcomes in the COVID-19 cohort. Our mortality analysis showed a nonsignificant difference in the rate of in-hospital mortality in patients receiving HCQ group compared to those in the control group. It should be noted, however, that there was a two-fold higher risk of death in the HCQ arm. These findings are in line with a previous study by Magagnoli et al [16] that also reported a three times higher odds of death in patients receiving HCQ. Following this, another French study consisting of 181 patients with diagnosed COVID-19 pneumonitis reported that HCQ use was of no benefit [17]. In terms of mortality, the results of our study are similar to the recently published preliminary results of the RECOVERY trial in which patients were randomized between the HCQ group (n=1542) and usual care group (n=3132). There was no significant difference in 28-day mortality between the two groups (hazard ratio 1.11, 95 % CI 0.98-1.26; *P*=.10) [3]. Following these results, the FDA revoked the EUA for use of HCQ in COVID-19 patients on June 15, 2020 [18].

The most debilitating complication of SARS-CoV-2 infection is acute respiratory failure, necessitating the use of IMV and other concurrent resource-intensive tools in critical care units [15]. Previous studies have reported mixed results, showing 11% to 44% use of IMV in patients receiving HCQ and TCZ [13,19,20]. Magagnoli et al [17] included sicker patients, who were more likely to receive HCQ on compassionate grounds and hence were more prone to have adverse outcomes and death, calling into question its reliability. By contrast, our analysis adjusted the pooled estimate of IMV requirement in both HCQ and TCZ groups by identifying major potential confounders such as baseline comorbidities and other medications used during the hospital stay. By demonstrating a nonsignificant trend in all the above-mentioned outcomes, we recommend against the routine use of HCQ and TCZ in patients with COVID-19.

### Limitations

The limitations of our study should be considered when interpreting the results. Due to the retrospective nonrandomized nature of the study, a causal relationship could not be established. Although the overall findings were adjusted for covariates including baseline comorbidities and medications, the impact of unmeasured confounders, such as initiation of several complementary therapies at the treating physician’s discretion, could not be determined. Based on our clinical experience, the average duration of any therapy for COVID-19 was less than 7 days; therefore, we chose to use laboratory values from day 1 and day 7. However, given the variable frequency of laboratory specimen collection, it is not possible for us to ascertain if these values truly represented pre- and posttreatment values accurately in all cases. The patients who received TCZ were mainly selected based on the availability of the drug (which was in short supply intermittently during the time frame of our study), and these patients were sicker with lower PaO2/FiO2 ratios. Moreover, by excluding patients still in the hospital, the case fatality ratio in our study cannot reflect the true mortality of COVID-19. Our study did show a trend of beneficial events in terms of the point estimate of the pooled effect size. However, there was an overlap in the confidence intervals and broad confidence intervals indicating that our study was underpowered to reach the level of significance. Although we adjusted the outcomes against demographics and underlying comorbidities, neither did we evaluate the contribution of underlying comorbidities to COVID-19 mortality via propensity score matching nor did our study evaluate the potentially harmful effects of these medications. We believe that a large-scale study will determine the true merits of these medications and will also reveal the potentially harmful outcomes of these medications. Many questions remain open, however. By adjusting the adult patients with the confirmed disease, we believe our population is the representative of the real-world cohort.

### Conclusion

HCQ and TCZ use was not associated with a reduction in endpoints of in-hospital mortality, upgrade to medical ICU, need for IMV, acute renal failure necessitating dialysis, or discharge from the hospital. Although there was a significant reduction in CRP level on day 7 of hospitalization in the patients receiving TCZ, the lack of improvement in hard clinical outcomes suggests that large-scale randomized controlled trials are needed to evaluate the efficacy of these drugs.

## Abbreviations

ANOVA: analysis of variance
aOR: adjusted odds ratio
CAD: coronary artery disease
CKD: chronic kidney disease
COPD: chronic obstructive lung disease
CRP: C-reactive protein
DM: diabetes mellitus
EUA: emergency use authorization
FDA: Federal Drug Administration
HCQ: hydroxychloroquine
HTN: hypertension
ICU: intensive care unit
IL-6: interleukin-6
IMV: invasive mechanical ventilation
RT-qPCR: real-time qualitative polymerase chain reaction
TCZ: tocilizumab
uOR: unadjusted odds ratio
